# AMPK activates DAPK2 to promote autophagy

**DOI:** 10.18632/oncotarget.25842

**Published:** 2018-08-03

**Authors:** Ruth Shiloh, Adi Kimchi

**Affiliations:** Department of Molecular Genetics, Weizmann Institute of Science, Rehovot, Israel

**Keywords:** DAPK2, AMPK, autophagy

DAPK2 (Death-associated protein kinase 2; also named DRP-1) is a member of the DAPK family of proteins: death associated Ser/Thr kinases that belong to the calmodulin (CaM)-regulated kinase superfamily [1, 2]. DAPK2 mediates a range of cellular processes [3], but special interest was focused in recent years on its role in autophagy [4-6]. DAPK2 activity in cells is tightly regulated by several inhibitory mechanisms. In the absence of CaM, the CaM auto-regulatory domain of DAPK2 binds to its catalytic cleft, blocking access of exogenous substrates. Autophosphorylation of Ser308 within the CaM auto-regulatory domain stabilizes its docking in the catalytic cleft, thus reinforcing this inhibitory mechanism. Binding of CaM to the CaM auto-regulatory domain, as well as Ser308 dephosphorylation, relieve this inhibition by promoting release of the CaM auto-regulatory domain from the catalytic cleft [7]. Another level of inhibition is provided by homodimerization of DAPK2, which further blocks its catalytic site [8]. Additionally, phosphorylation of one or more of the last four amino acids of the protein (SSTS) inhibits DAPK2 activity by mediating 14-3-3 binding [9].

In our recent study, we identified AMPK as a positive upstream regulator of DAPK2 [10]. AMPK is a metabolic sensor and a key regulator of energy homeostasis in eukaryotic cells. Once activated, it acts to suppress anabolic pathways and promote catabolic pathways, such as autophagy. We found that AMPK phosphorylates DAPK2 on a single site, Ser289, thus elevating its catalytic activity (Figure 1). Notably, we found that this single phosphorylation also affects significant biochemical properties of DAPK2. It induces ablation of the inhibitory Ser308 autophosphorylation and reduction in homodimerization, two activating events that were known to be induced by binding of CaM to the CaM-binding helix. Thus, Ser289 phosphorylation in fact functionally mimics CaM binding. We hypothesize that this single phosphorylation can induce these effects due to its critical location. According to the known crystal structure of DAPK2, Ser289 is located in a short loop between the catalytic and CaM auto-regulatory domains. This loop has been proposed to act as a flexible hinge allowing a swing-out motion of the CaM-binding helix, thus exposing the catalytic cleft. Introduction of a negative charge in the form of a phosphate group at this critical location may change the protein’s conformation in a way similar to the effect of CaM binding. By doing so, Ser289 phosphorylation by AMPK provides an alternative mode of DAPK2 activation that is CaM-independent. These new findings provide a unique concept in the field of CaM-regulated kinases, in which a single phosphorylation can bypass the dependence on the canonical mechanism of activation by calcium signaling. Interestingly, metabolic stress and calcium signaling seem to be mutually exclusive pathways for DAPK2 activation, as we found that a Ser289 phospho-mimicking mutation reduces DAPK2’s affinity to CaM.

**Figure 1:**
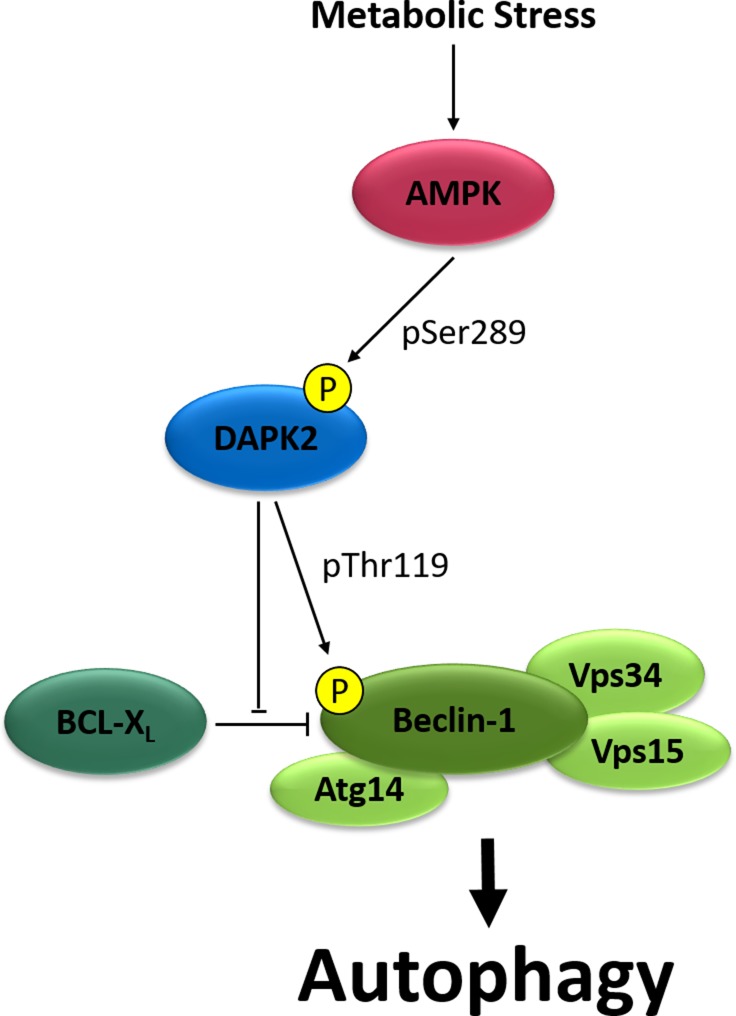
Schematic representation of the AMPK-DAPK2 pathway AMPK is activated in response to metabolic stress, phosphorylates DAPK2 on Ser289 and elevates its catalytic activity. DAPK2 phosphorylates Beclin-1 on Thr119, causing dissociation of its inhibitor BCL-X_L_ and promoting autophagy induction.

Notably, Ser289 phosphorylation by AMPK is specific to DAPK2 as this mode of regulation is not shared with DAPK1. Despite the fact that this residue is located in a region that is homologous between the two kinases and is conserved in both, we found that it is not phosphorylated in DAPK1 upon AMPK activation

in cells. However, this site was previously found to be phosphorylated in DAPK1 by RSK, a member of the MAPK family [11]. This difference in regulation by upstream signaling pathways suggests different roles for these two similar kinases, as both may be activated in cells in response to different stimuli.

The identification of AMPK as a novel upstream regulator of DAPK2 is the first evidence indicating DAPK2 can be activated by metabolic stress, and links DAPK2 to metabolic-stress-induced autophagy. Our work further links DAPK2 to autophagy by the identification of Beclin-1 as a novel substrate of DAPK2. Beclin-1 is part of a multi-protein complex that includes also the lipid kinase Vps34, Vps15 and Atg14 and is responsible for the production of PI(3)P at the autophagosome assembly site, which is the first step in autophagosome assembly. A previous screen for protein-protein interactions within the cell death network identified Atg14 as a binding protein of DAPK2 [9], implying a functional link to this complex. DAPK2 phosphorylates Beclin-1 on Thr119, causing the dissociation of its inhibitor BCL-X_L_ and thus promoting autophagy induction (Figure 1). Ser289 phosphorylation of DAPK2 by AMPK elevates DAPK2’s ability to phosphorylate Beclin-1, and depletion of DAPK2 reduces autophagy induction in response to AMPK activation in cells. Thus, the AMPK-DAPK2 axis plays an important role in autophagy induction in response to metabolic stress.

## References

[1] Inbal B (2000). Mol Cell Biol.

[2] Shiloh R (2014). Apoptosis.

[3] Geering B. (2015). Int J Biochem Cell Biol.

[4] Ber Y (2015). Cell Death Differ.

[5] Soussi H (2015). Diabetes.

[6] Chi HC (2016). Autophagy.

[7] Shani G (2001). EMBO J.

[8] Simon B (2016). Structure.

[9] Gilad Y (2014). Cell Rep.

[10] Shiloh R (2018). Nat Commun.

[11] Anjum R (2005). Curr Biol.

